# Reported co-infection deaths are more common in early adulthood and among similar infections

**DOI:** 10.1186/s12879-015-1118-2

**Published:** 2015-10-06

**Authors:** E. C. Griffiths, A. B. Pedersen, A. Fenton, O. L. Petchey

**Affiliations:** Department of Animal and Plant Sciences, University of Sheffield, Alfred Denny Building, Western Bank, Sheffield, S10 2TN UK; Department of Entomology, North Carolina State University, Campus Box 7613, Gardner Hall, Raleigh, NC 27695-7613 USA; Centre for Immunology, Infection and Evolution & Institute of Evolutionary Biology, School of Biological Sciences, Ashworth Labs, University of Edinburgh, Kings Buildings, Charlotte Auerbach Road, Edinburgh, EH9 3FL UK; Institute of Integrative Biology, University of Liverpool, Liverpool, L69 7ZB UK; Institute of Evolutionary Biology and Environmental Studies, University of Zürich, Winterthurerstrasse, Zürich, 8057 Switzerland

## Abstract

**Background:**

Many people have multiple infections at the same time, but the combined contribution of those infections to disease-related mortality is unknown. Registered causes of death offer a unique opportunity to study associations between multiple infections.

**Methods:**

We analysed over 900,000 death certificates that reported infectious causes of death. We tested whether reports of multiple infections (i.e., co-infections) differed across individuals’ age or sex. We also tested whether each pair of infections were reported together more or less often than expected by chance, and whether this co-reporting was associated with the number of biological characteristics they had in common.

**Results:**

In England and Wales, and the USA, 10 and 6 % respectively of infection-related deaths involved co-infection. Co-infection was reported reported most often in young adults; 30 % of infection-related deaths among those aged 25–44 from the USA, and 20 % of infection-related deaths among those aged 30–39 from England and Wales, reported multiple infections. The proportion of infection-related deaths involving co-infection declined with age more slowly in males than females, to less than 10 % among those aged >65. Most associated pairs of infections co-occurred more often than expected from their frequency of being reported alone (488/683 [71 %] in the USA, 129/233 [55 %] in England and Wales), and tended to share biological characteristics (taxonomy, transmission mode, tropism or timescale).

**Conclusions:**

Age, sex, and biologically similar infections are associated with death from co-infection, and may help indicate patients at risk of severe co-infection.

**Electronic supplementary material:**

The online version of this article (doi:10.1186/s12879-015-1118-2) contains supplementary material, which is available to authorized users.

## Background

Infectious diseases cause 25 % of human mortality worldwide; in 2008, respiratory infections caused 4.26 million deaths, diarrhoea caused 2.16 million deaths, and HIV/AIDS caused over 2 million deaths [1]. However, the role of co-infection (more than one simultaneous infection in an individual) in this mortality is unknown. Some co-infections are known to cause death; for example HIV-tuberculosis co-infection caused 350,000 deaths worldwide in 2008 [2], and bacterial pneumonia increases the risk of death from influenza [3]. While some co-infections are not detrimental, most papers report a negative effect of co-infection on human health [4]. Despite previous reports of negative health effects, we know little about the characteristics of people who died from co-infection.

A key question is whether deaths due to co-infection are predictable, and what factors influence this. Demographic characteristics (i.e., age, sex) of individuals could be important determinants of whether co-infection is reported on a death certificate. Older people and males are typically more susceptible to infectious disease than younger people and females [5–7]. There is evidence of age and sex biases for certain co-infections; most measles deaths are from viral or bacterial co-infection in young females [8], whereas sepsis deaths are higher in males than females [9]. Whether deaths from many different co-infections generally differ by age or sex is unclear.

Characteristics of the infecting organisms could also underlie associations among reported infections. We hypothesise that biologically similar pairs of infections co-occur more often than expected. For example, taxonomically similar infections may coinfect due to similar life cycles, targeted organs, or antigens [10, 11]. Shared transmission routes may promote co-infection, e.g., bloodborne viral infections among injecting drug users [12]. Similarly, chronic infections in the same body part may exacerbate morbidity (e.g., hepatitis viruses A and C [13]). An alternate hypothesis is that antagonistic interactions are stronger between more similar infections, and thus would be found together less often.

The characteristics of people who died with co-infections can be studied using causes of death reported on death certificates. These data offer a general description of co-infection-associated death in humans, providing broad context for other co-infection research, and enable tests of specific, public-health relevant hypotheses about co-infection. Here, we use death certificates to test for relationships between co-infection-related deaths and individual age and sex, and explore how similarities in biological characteristics (taxonomy, transmission, tropism, and timescale) related to associations between reported infections.

We gathered data on reported infectious causes of death across a recent four-year time period in England and Wales, and the USA. These data offer a novel opportunity to study how infections associate with one another at death, and the contribution of characteristics of both the individual and the infections. We address three hypotheses: (i) the proportion of infection deaths attributed to multiple infections differs by age and sex, (ii) the frequency of pairs of infections co-occurring on death certificates differs from that expected from the occurrence of each infection alone, and (iii) the frequency of co-occurrence of each pair of infections on death certificates increases with similarity in terms of taxonomic group, transmission route, tropism, and timescale.

## Methods

Death certificates in England and Wales report one underlying cause of death and up to 15 contributory factors, following the International Classification of Diseases (ICD [14]). We used 139,459 death certificates from 2005 to 2008 in England and Wales that reported at least one infection and followed ICD-10. In England and Wales 2005–08 was the longest recent time period within which ICD codes were interpreted consistently. In the USA, one main and up to 20 extra causes of death are listed on death certifications following ICD-10. There were 816,390 death certificates from 2005 to 2008 in the USA that reported at least one infection. By infection we mean a type of infectious disease classified in ICD, not necessarily a particular pathogen.

In ICD-10, one infection code explicitly indicates co-infection (‘B20’, which denotes other infections arising from HIV infection). Other co-infections are indicated by multiple infections reported on the death certificate. Hereafter, the term “single infection death” indicates death certificates with only one infection reported, and “co-infection death” indicates death certificates with more than one infection reported.

Data for England and Wales were obtained from the Office for National Statistics. Other data provided were sex and age at death (eight age categories: 0–19, 20–29, 30–39, 70–79, 80+). Other information, (i.e., exact age, date and place of death, higher resolution ICD codes) were removed by the Office for National Statistics to prevent identification of individuals.

Data for the USA were downloaded from http://www.nber.org/data/multicause.html. For comparability with England and Wales we ensured similar ICD coding, and roughly decadal categories, while also keeping higher resolution data available among children (<1, 1–4, 5–14, 15–24, 25–34, 75–84, 85+). We also used data on place of death (e.g., patients in hospital, hospice, or nursing home) to test whether the patterns were consistent among inpatients with access to treatment before death.

### Ethical approval

We use data from public agencies in the US and the UK that relate to deceased humans. We were exempt from requiring ethical approval to undertake our study because the individuals were not living when the data were gathered (US federal regulations 45 CFR 46.102f Protection of Human Subjects 2009). We also worked with the Office for National Statistics to ensure that data they supplied and the results that we report herein did not disclose personal or sensitive data relating to living persons (Freedom of Information Act 2000 c. 36 II section 40(3)(a)).

### Statistical analyses

(i) Age, sex, and co-infection death
  Associations between age, sex, and co-infection death were modelled using generalised additive models (GAM) with two predictor variables: sex (two level factor; male and female) and age (eight level factor for England and Wales, eleven level factor for the USA), and the interaction between age and sex. This analysis is similar to a logistic (i.e., binomial) regression where the response variable is the number of “successes” (co-infection death) and “failures” (single infection death) with binomial error structure and logit link, except GAM allows for non-linear relationships between co-infection and age (e.g., [15]). We used the Akaike Information Criterion (AIC), where a lower AIC indicates a more informative model, to determine which terms should remain in the model. We started with a saturated model and proceeded to drop interactions and then main effects if their deletion reduced AIC.

(ii) Associations between pairs of infections
  For each pair of infections we tested whether the number of deaths involving both infections was different from that expected in the absence of any association using a Chi-squared test. Every co-occurring infection was included in this analysis such that a death certificate reporting three infections would have contributed three pairs. The residuals from this analysis were used to quantify the disparity between the observed and expected frequencies of co-infection death; a negative residual indicated fewer co-infection deaths than expected, while a positive residual indicated more than expected. To account for infections being reported with different frequency, we report Pearson standardised residuals [16].

(iii) Are biologically similar infections associated with co-infection death?
  We tested whether the measured associations between each pair of infections were related to the biological similarity between them, based on four characteristics: taxonomy, transmission, tropism, and timescale (see Additional file 1: Supplementary Information S2 for details). For each infection we gathered data on each characteristic using PubMedHealth [17], a human infection database [18], and an RNA virus database [19].
  Pairwise similarity between each pair of infections was calculated as the number of matching characteristics (zero – four). We used a Mantel test [20] to measure the correlation coefficient between the matrix of pairwise biological similarities and the matrix of pairwise associations on death certificates (standardised Chi-squared residual values).
  All analyses were done in R version 3.1.0 [21], including using the mgcv package to fit GAMs and calculate AIC [22] and the ade4 package for Mantel tests [23].

## Results

From the 9,769,977 death certificates in the USA between 2005 and 2008, 732,079 (7.5 %, 369,646 female and 362,433 male) attributed death to one infection, while 84,311 (0.9 %, 33,513 female and 50,798 male) attributed death to multiple infections. From the 2,028,734 death certificates in England and Wales between 2005 and 2008, 130,758 (6.4 %, 72,080 female and 58,678 male) attributed death to one infection, while 8695 (0.4 %, 4623 female, 4072 male) attributed death to multiple infections.

**Fig. 1:**
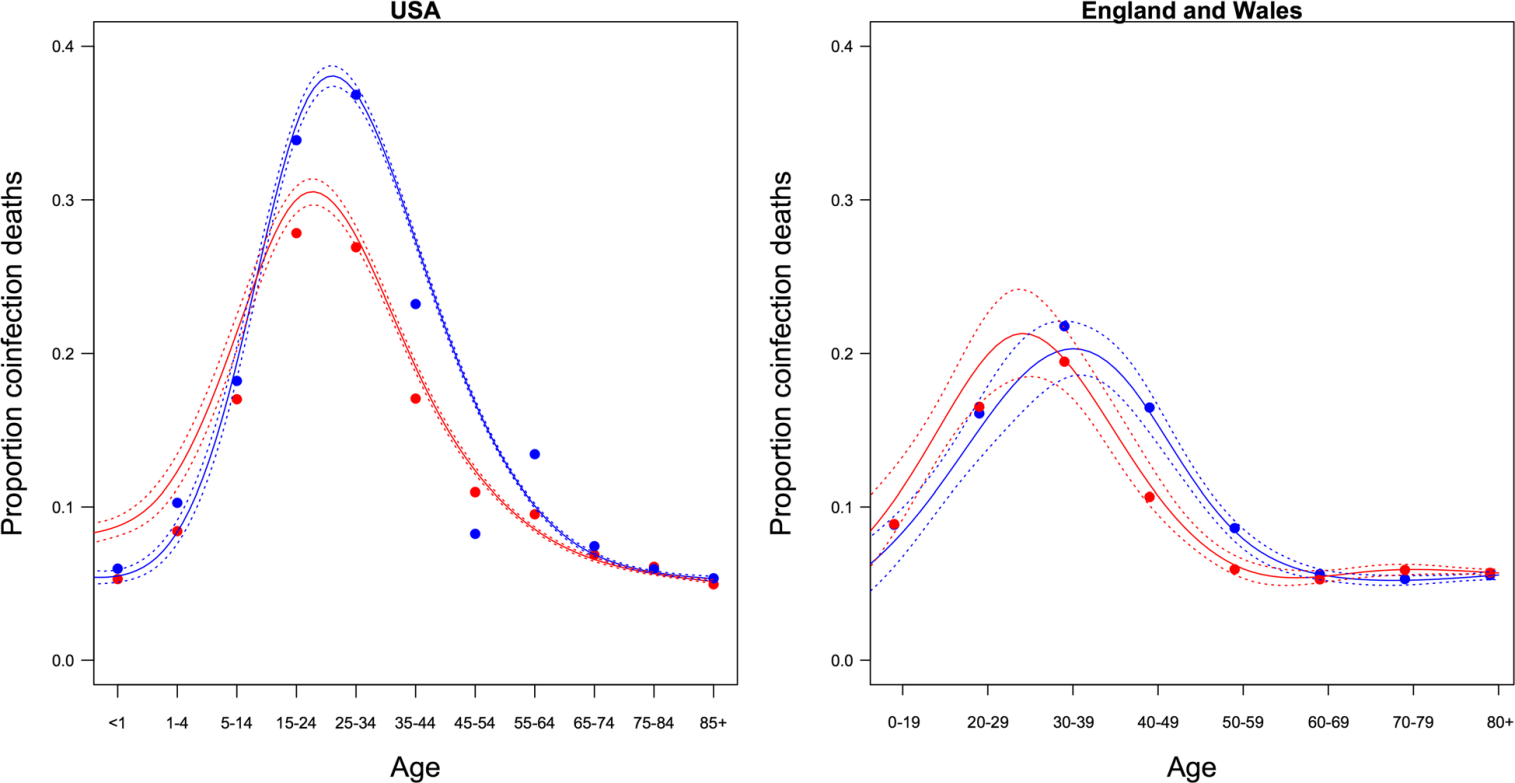
Proportion of infection-related deaths reported due to co-infection in the USA (left) and England and Wales (right). Points are the observed proportions of co-infection death among death certificates reporting at least one infection. Solid lines are the fitted binomial GAM (female = red, male = blue). Dashed lines are two standard errors above and below the fitted values
  To test the sensitivity of this result to our analytical methods we tested for associations between co-infection death, age, and sex using other settings; in all instances we found omission of the age:sex interaction to increase AIC by at least 25 (Additional file 1: Table S1).

**Fig. 2:**
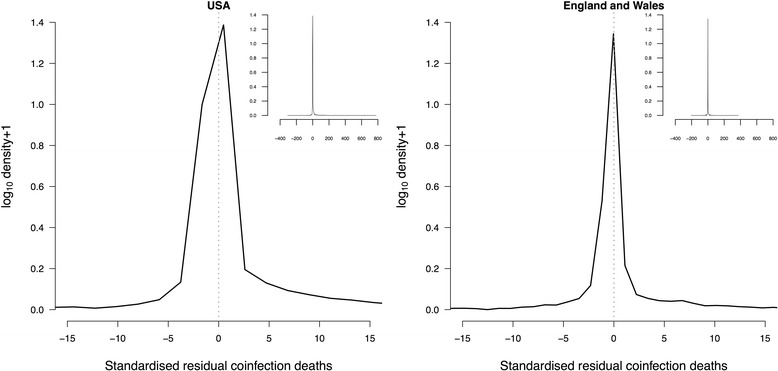
Distribution of association of pairs of infections on death certificates in the USA (left) and England and Wales (right). Solid black lines are the density curves of standardised residuals for the number of co-infection deaths from Chi-squared tests. Vertical dashed grey line at zero residual. Inset shows the whole distribution
  Nevertheless, the strongest associations tended to be positive. For example, the proportion of pairs with residuals greater than 5 is 5.05 % in the USA, and less than −5 is 1.24 %. And for England and Wales these proportions are 2.81 and 1.55 %. The longer positive tails on the distribution were also found for non-standardised and adjusted residuals, particularly for the USA data (Additional file 1: Figures S1 and S2).

(i) Age, sex, and co-infection death
  In the USA, and England and Wales, the proportion of deaths attributed to co-infection rose from the youngest age classes, peaking during adulthood (fitted value ± se: 0.386 ± 0.004 for males at 30 and 0.294 ± 0.005 for females at 28 in the USA and 0.205 ± 0.010 for males at 40 and 0.209 ± 0.013 for females at 35 in England and Wales), and then subsiding to low levels in the older age categories (Fig. 1, binomial GAM with age spline by sex, lowest at age 85 for males [0.056 ± 0.001] and 85 for females [0.059 ± 0.001] in the USA and at age 75 for males [0.052 ± 0.002] and 67 for females [0.055 ± 0.001] in England and Wales). The peak age of co-infection death was later in males than females (Fig. 1, removing the age:sex interaction increased AIC by 459 [USA] and 39 [England and Wales]).

(ii) Associations between pairs of infections
  Of 9453 possible pairings from 138 infections reported on death certificates in the USA, 1067 (11 %) co-occurred on death certificates. Of 4560 possible pairings from 96 infections reported on death certificates in England and Wales, 366 (8 %) co-occurred on death certificates.
  Most pairs co-occurred less often than expected, indicated by the positive skew in Chi-squared residuals (Fig. 2, USA: 91.2 % had a negative standardised residual; England and Wales: 94.2 % had a negative standardised residual).

(iii) Are biologically similar infections associated with co-infection death?
  There were 3501 pairs of associated infections reported in both the USA and England and Wales. Standardised residuals in both countries were positively correlated (Additional file 1: Figure S3, *r* = 0.32, df = 3499, 95 % CI 0.303–0.345). Hence, most of these pairs of infections (3089/3501, 88.2 %) had the same direction of association in both countries and we have greater certainty over their co-occurrence. Pairs with the strongest negative residuals in both countries were A41 Other septicaemia and A04 Other bacterial intestinal infections (standardised residual −143 in England and Wales and −206 USA), A41 and B18 Chronic viral hepatitis (−307 and −32), A41 and A49 Bacterial infection of unspecified site (−125 and −64), and A41 and B24 Unspecified HIV disease including AIDS (−142 and −31; see http://figshare.com/preview/_url/1328406/project/3684 for residuals of all pairs in both countries). We used these pairs with similar co-occurrence in both countries to investigate how co-occurrence on death certificates was associated with biological similarity between each pair of infections.
  The standardised chi-squared residual for a pair of infections was positively correlated with the number of shared biological characteristics (Mantel test with 100 repetitions simulated mean ± 2sd: 0.24 ± 0.17). In other words, infections that co-occurred more often than expected tended to have more characteristics in common. Co-occurring pairs tended to share each characteristic (separate Mantel tests with 100 repetitions: tropism 0.19 ± 0.14, transmission 0.12 ± 0.11, taxonomy 0.15 ± 0.13, timescale 0.18 ± 0.17, Additional file 1: Figure S4).
  To test the sensitivity of this result to our analytical methods we also tested for associations using data for each country alone, and using linear regression between the number of shared characteristics and the Chi-squared residual. In all instances we found a strength of association whose confidence intervals did not overlap zero (Additional file 1: Supplementary Information S4).

## Discussion

Humans can get many different co-infections, but treatment guidelines only exist for a few specific combinations (e.g., HIV and hepatitis C). Co-infection morbidity has also been studied within certain cohorts (e.g., 5–16 year olds, [15]), and is often reported to be worse than single infections [4]. However, the occurrence of co-infection in death across age and sex cohorts has, to our knowledge, never been studied before. Our results indicate that (i) co-infection death may be more common in early adulthood, but it is not known whether younger adults are more susceptible to co-infection per se, or more susceptible to fatal co-infection. We also found that (ii) pairs of infections with strong positive association on death certificates tended to co-occur more often than those with strong negative associations. This suggests that medical care of severely ill patients with some co-infections can be problematic. Finally, (iii) co-occurrence on death certificates was positively related to biologically similarity. Better understanding of these biological interactions may help efforts to predict and combat co-infection mortality. We discuss the factors that may contribute to these patterns, before considering implications for treatment, limitations of the data, and future research needs.

### Possible causes

The early-to-mid adulthood peak in co-infection death contrasts with theories that the immune response declines in old age [5], and with non-infectious diseases where comorbidities increase with age [24]. This could be explained by individuals being more susceptible to death from one infection in old age, either because they are frailer as their bodies deteriorate through oxidative damage [25], or the infection coincides with non-infectious causes of death that are more common with age, like cancer [26]. Alternatively, young adults are more prone to severe immunopathologies following infection: critically ill patients with influenza A(H1N1) tended to be 20–30 years old [27], and the added physiological stress of co-infection might make death more likely. Another possibility is that more effort is made to find infections in critically ill young adults than for older patients. We are not aware of evidence that biased medical practices also contribute alongside the physiological factors mentioned above, but this is a possibility that could be examined further.

Reasons for males being at higher risk of infection than females include behaviours that put them at greater risk of infection, or physiological reasons, such as sex hormones, that make them more susceptible to severe disease once infected [7, 28]. Our data do not enable us to distinguish which of these mechanisms may have played a role. If males undertake riskier behaviour, have higher testosterone in early adulthood, or are less likely to visit the doctor when ill this may explain why the sex difference appears around the peak of the distribution (Fig. 1).

### Treatment implications

Our results suggest that co-infection treatment guidelines could be based on synergistic interactions between infections. Most possible pairs of infections co-occurred on death certificates at a frequency expected from their occurrence alone. We suggest that the unassociated pairs of infections could be excluded from efforts seeking to identify severe co-infections.

Around 1 in 20 possible pairs were associated and tended to co-occur more often than expected. Positively associated pairs of reported co-infections included: mycobacteria and HIV, viral hepatitis co-infection, and cytomegalovirus and pneumocystis. While these similar pairings were often reported together, associations were context dependent; they were negatively associated with other infections, including mycobacteria and infectious bloody diarrhoea, pneumocystis and sequelae of tuberculosis, and viral hepatitis and Zoster virus infection. The direction of association is therefore not consistent for the same infection, and so treatment guidelines should not be based solely on the identity of one constituent infection. Whether the relatively weak correlations are clinically meaningful remains a debatable point, but on a population scale, across hundreds of thousands of deaths, the results suggest that it may be important to public health and worthy of further investigation. The biological similarity of associated pairs could be an important consideration when assessing the potential severity of a given co-infection.

### Data quality and limitations

Studies based on reported data must consider potential biases. In our dataset there may be underreporting of co-infection death on death certificates if infectious disease was undetected, wrongly deemed not to have contributed to death, or were not reported using multiple codes. Poor reporting of causes of death was a problem in the UK in the 1990s [29]. There have since been legal and educational reforms [30], and death certificate data have been audited by the Center for Disease Control and the Office for National Statistics. Using multiple infectious causes as indicators of co-infection probably underestimates the true number of co-infection deaths. One could hypothesise that certain types of infections, such as those detected by the same test, with similar tropism, of high severity, might be more likely to be diagnosed. However, from death certificates alone we are unable to examine whether behaviour or diagnostic techniques may have played a role. We have no evidence of systematic bias that could have generated the patterns we found, but we encourage further broad scale analyses of co-infection to help establish the key factors of the individual and their infections that can best guide treatment. Our conclusions are robust to the complexity of model fitted (Additional file 1: Supplementary Information S1), measure of association used (Additional file 1: Supplementary Information S2), country and method for analysing biological similarity (Additional file 1: Supplementary Information S3 and S4), ambiguity in ICD-10 codes (Additional file 1: Supplementary Information S5), and inpatient status (Additional file 1: Supplementary Information S6). Therefore, we are confident that we describe genuine patterns.

Other limitations to the secondary data available include: an inability to distinguish certain pathogens within the ICD-10 disease codes, severity of disease not necessarily corresponding with both infections being of the same timescale, and the age categories reported being somewhat arbitrary and not matching physiological changes like puberty.

### Further research

Causes of death are associated with various factors including healthcare, socioeconomic status, family structure, geography, behaviour, physiology, or infectious dose. Determining what factors affect causes of death using national observational data alone is difficult. Co-infection death needs to be assessed in other time periods and countries.

The patterns we described could be attributed to biological interactions, or an artefact of the relative prevalence of the infections among at-risk populations. To disentangle the two we need data on co-infection prevalence. While we have some evidence that the number of reported infection deaths is not correlated with reported infections in England and Wales (Additional file 1: Supplementary Information S7), this was only for a subset of infections, and we could not find data on prevalence for most co-infections in our dataset.

## Conclusions

We studied co-infection mortality across infections, from viruses to helminths, and found several patterns not previously described: (i) the positive skew among thousands of pairs of reported infections, (ii) the distribution of co-infection deaths across age and sex cohorts, and (iii) the tendency for biologically similar infections to associate positively with reported co-infection mortality. Having described these broad scale patterns we can now put particular co-infection studies in context, and help target healthcare appropriately to prevent co-infection death.

## Additional file

Additional file 1: 
**Supplementary analyses including table S1 and supplementary figures S1 to S10.** (DOCX 3588 kb)
