# Supplementary material for: Reported co-infection deaths are more common in early adulthood and among similar infections
Source: BMC Infect Dis. 2015 Oct 6;15:411. doi: 10.1186/s12879-015-1118-2 (PMC4595177; doi:10.1186/s12879-015-1118-2)
Supplement: Additional file 1: — Supplementary analyses including table S1 and supplementary figures S1 to S10. (DOCX 3588 kb) [file 12879_2015_1118_MOESM1_ESM.docx]

Reported co-infection deaths are more common in early adulthood and among similar infections: Supplementary Information

Supplementary data and code have been publicly uploaded to Figshare:

http://figshare.com/account/projects/3684

1. Alternative analyses of age, sex, and proportion of infectious disease deaths involving coinfection

Table S1: Results of analysis of deviance tests and Akaike Information Criterion comparisons on different statistical models of the proportion of deaths from coinfection in a given country’s data.

|  | **GAM** | | | **Logistic regression (binomial glm)** | | |
| --- | --- | --- | --- | --- | --- | --- |
|  | Knots in age spline | Change in deviance when sex removed from age spline | Change in AIC when sex removed from age spline | Degree of polynomial | Change in deviance when age:sex interaction removed from highest order polynomial | Change in AIC when age:sex interaction removed from highest order polynomial |
| **USA** | 3 | 870.97 | 868 | 1 | 372 | 370 |
|  | 4 | 525.71 | 521 | 2 | 1033.7 | 1029 |
|  | 5 | 409.07 | 403 | 3 | 485.59 | 479 |
|  | 6 | 467.28 | 459 | 4 | 600.26 | 592 |
|  | 7 | 501.83 | 492 | 5 | 528.19 | 518 |
|  | 8 | 523.51 | 512 | 6 | 529.11 | 517 |
| **England and Wales** | 3 | 27.941 | 26 | 1 | 34.849 | 33 |
|  | 4 | 28.72 | 25 | 2 | 41.488 | 37 |
|  | 5 | 47.391 | 42 | 3 | 42.418 | 36 |
|  | 6 | 45.912 | 39 | 4 | 54.2 | 46 |
|  | 7 | 45.962 | 38 | 5 | 51.938 | 41 |
|  | 8 | 45.966 | 37 | 6 | 53.869 | 42 |

1. Additional Chi-squared residual results

a) USA

Fig. S1 Density of three types of residual for reported coinfection deaths from significant Chi-squared tests for the USA. Top=raw residual, middle=standardized residual (corresponding with main manuscript), bottom=adjusted residual. See Agresti (2012) for details on residuals.

b) England and Wales

Fig. S2 Density of three types of residual from significant Chi-squared tests of reported coinfection deaths for each pair of infections for England and Wales. Top=raw residual, middle=standardized residual (corresponding with main manuscript), bottom=adjusted residual. See Agresti 2012 for details on residuals.

1. Standardised residuals in different countries

Fig. S3 Standardised Chi-squared residuals for 3501 pairs of infections reported together on death certificates in the USA and in England and Wales. We attribute the

3. Methods for gathering data on biological similarity

Taxonomic categories were: viruses, bacteria, fungal parasites, protozoa, and helminths. Transmission categories were: contaminated food/water, inhalation, insect bites, open wounds, animal contact, skin contact, sexual contact, or environmental pathogens. Tropism categories were: neuronal, respiratory, circulatory, gastrointestinal, genital, skin, glandular, or multi-organ. Timescale was recorded as either acute or chronic. We excluded infections with ambiguous timescales like *Chlamydia*, Q fever, or *Nocardia*; multiple tropisms, or unspecified taxonomy, e.g. A09 (“diarrhoea of presumed infectious origin”).

4. Further analyses of biological similarity

1. Number of shared characteristics in associated and unassociated pairs

Fig. S4 Standardised residual for coinfection pairs in the USA, and whether or not they shared four biological characteristics, for those pairs with significant associations (beyond 95% CI, panels A-D) and no association (within 95% CI, panels E-H).

b) Country-specific Mantel tests

In the main text we reported Mantel Tests of correlation between biological similarity and standardized Chi-squared residuals of pairs of coinfections reported in England and Wales and the USA.

We repeated these analyses separately by country. For the USA there was a positive association for the number of shared characteristics (Mantel test with 100 repetitions r=0.13), and each characteristic in turn (Fig. S4, Mantel tests with 100 repetitions: Tropism r=0.09, Timescale r=0.14, Transmission r=0.02, Taxonomy r=0.10). For England and Wales there was a significant positive correlation between pairwise strength of association on death certificates and the number of shared biological characteristics (Mantel test with 100 repetitions: r=0.74) and tended to share each characteristic analysed separately (Fig. S5, Mantel tests with 100 repetitions: Tropism r=0.62, Transmission r=0.65, Taxonomy r=0.69, Timescale r=0.63).

Fig. S5 Standardised residual for coinfection pairs in the USA, and whether or not they shared four biological characteristics.

Fig. S6 Standardised residual for coinfection pairs in England and Wales, and whether or not they shared four biological characteristics.

c) Linear regression

For pairs that had standardized residuals with the same direction in both countries, we also used linear regression to test for significant interactions among the four biological characteristics. We started with a saturated model: √(Pearson residual) ~ tropism * transmission * time * taxon where the predictors are binary variables of whether or not the pair of infections shared that characteristic. We deleted the interactions and then the main effects of any variables whose exclusion reduced AIC by at least two points.

The optimal model had a relatively large F-statistic (*F*_9,1035_=8.03):

√(Pearson residual) ~ tropism + taxon + time + transmission

+ taxon:transmission + taxon:tropism

+ transmission:tropism + taxon:time

+ taxon:transmission:tropism

No main effect or two-way interaction had a standard error less than its coefficient and the optimal model only accounted for 6% of the variance (R^2^=0.065). However, there was a strong three-way interaction whereby coinfections sharing the same tropism, taxonomic group, and transmission route had higher Chi-squared residuals (β=1.67, se=0.59), indicating that these characteristics together are associated with co-occurrence on death certificates.

We also used linear regression to test whether the standardized Chi-squared residuals of pairwise coinfection death of the same direction in England and Wales and the USA increased the more characteristics a pair had in common. We used the same method for model selection as above. With each additional shared characteristic, the square root standardized residual of coinfection death increased by 0.07 (s.e. 0.02, Fig. S7). While this model has a relatively large F-statistic (*F*_1,3087_=11.8), the wide distribution of residuals means it has an R^2^ value of 0.003.

 Fig. S7 Square root transformed standardized Chi-squared residuals for coinfection death in the US and the number of shared biological characteristics for the 3089 pairs of infections that also had the same direction of residual in England and Wales.

5. Sensitivity to aggregation of ICD-10 codes

Some ICD-10 codes are caused by the same type of organism: *Mycobacterium* *tuberculosis* (A15-A19 and B90), *Treponema pallidum* (A50-A53 and A65), unidentified acute encephalitis (A85-A86), dengue (A90-A91), Varicella Zoster (B01-B02), and HIV (B20-24). We repeated our analyses to test whether our results were sensitive to aggregation of the ICD-10 codes by infecting organism.

In England and Wales the proportion of coinfection deaths in age and sex cohorts peaked among younger adults (Fig. S7A), there was a positive skew in co-occurrence (Fig. S7B), the number of shared biological characteristics was positively associated with co-occurrence on death certificates (Mantel test with 100 repetitions between standardized Chi-squared coinfection residuals and number of shared characteristics: r=0.19, Fig. S7C), and each of the four characteristics tested contributed to this (Tropism r=0.14, Transmission r=0.21, Taxonomy r=0.34, Timescale r=0.36).

In the USA the proportion of coinfection deaths in age and sex cohorts peaked among younger adults with a secondary peak for males (Fig. S8A), there was a positive skew in co-occurrence (Fig. S8B), the number of shared biological characteristics was positively associated with co-occurrence on death certificates (Mantel test with 100 repetitions between standardized Chi-squared coinfection residuals and number of shared characteristics: r=0.13, Fig. S8C), and each of the four characteristics tested contributed to this (Tropism r=0.06, Transmission r=0.10, Taxonomy r=0.13, Timescale r=0.12).

Fig. S7 Tests of the three hypotheses from the main manuscript using the England and Wales dataset and combining infectious causes of death involving the same pathogen. A: Proportions of death certificates that were coinfection deaths. Points are the observed proportions within that decadal age range by sex (female=grey, male=black), solid lines are the fit from a binomial gam P(multiple infection)=s(age):Sex. B: Density of standardized residuals from Chi-squared tests on deaths involving each pair of pathogens. C: Standardized Chi-squared residuals for each pair of pathogens against the number of biological characteristics they had in common.

Fig. S8 Tests of the three hypotheses from the main manuscript for the USA combining infectious causes of death involving the same pathogen. A: Proportions of death certificates that were coinfection deaths. Points are the observed proportions within that decadal age range by sex (female=grey, male=black), solid lines are the fit from a binomial gam P(multiple infection)=s(age):Sex. B: Density of standardized residuals from Chi-squared tests on deaths involving each pair of pathogens. C: Standardized Chi-squared residuals for each pair of pathogens against the number of biological characteristics they had in common.

6. Sensitivity to deaths among inpatients in the USA

Most of the death certificates from the USA reported death to have occurred in an inpatient (i.e. they had been admitted to a hospital, 625385/816390, 76.6%). The proportion of coinfection deaths in age and sex cohorts peaked in younger adults. There was a secondary peak where males exceeded females (Fig. S9A). From the Chi-squared tests pairs that co-occurred more often than expected outnumbered those co-occurring less often than expected (Fig. S9B). The number of shared biological characteristics was positively associated with co-occurrence on death certificates (Mantel test with 100 repetitions between two-way Chi-squared contingency test residuals and number of shared characteristics: r=0.27), and each of the four characteristics tested contributed to this (Fig. S9C, Tropism r=0.13, Transmission r=0.20, Taxonomy r=0.25, Timescale r=0.24).

Fig. S9 Tests of the three hypotheses from the main manuscript for the USA for only those deaths reported among inpatients. A: Proportions of death certificates that were coinfection deaths. Points are the observed proportions within that decadal age range by sex (female=grey, male=black), solid lines are the fit from a binomial gam P(multiple infection)=s(age):Sex. B: Density of standardized residuals from Chi-squared tests on deaths involving each pair of pathogens. C: Standardized Chi-squared residuals for each pair of pathogens against the number of biological characteristics they had in common.

7. Notifiable infections and coinfection death in England and Wales

One possibility is that more common infections are more likely to be reported as coinfections on death certificates. Using Spearman’s Rank we tested for correlation between the number of reported deaths in England and Wales and reported cases. A positive correlation would mean that infections frequently reported on death certificates were also frequent in the population. We obtained independent data on notifiable infections in England and Wales from 2005 to 2008. Notifiable infections are a group of infections deemed to be of national interest and were reported by doctors to the Health Protection Agency (now Public Health England). This dataset comprises the best data with national coverage on number of cases of a subset of infections.

There were 96 infectious causes of death on death certificates in England and Wales from 2005 to 2008, 13 of which were also notifiable infections. Ten notifiable infections did not appear on any death certificate, and 83 infectious causes of death were not notifiable infections. There was no significant relationship between the number of death certificates with a particular infectious cause reported and the number of notified cases of that infectious disease for either sex (Fig. S10, paired Spearman’s Rank correlation for males *ρ* = −0.14, df = 12, and for females *ρ* = −0.02, df = 12). Therefore, based on this analysis of a subset of infections, infection and coinfection mortality are not associated with reported cases. Factors that may cause deaths to be out of kilter with reported cases are differences in pathogen virulence, ease of diagnosis, and drug resistance.

Fig. S10 Number of reported deaths from the 13 infectious causes of death for which there was data on the number of reported cases in England and Wales for (A) females, and (B) males.
